# Healthcare Worker Contact Networks and the Prevention of Hospital-Acquired Infections

**DOI:** 10.1371/journal.pone.0079906

**Published:** 2013-12-30

**Authors:** Donald E. Curtis, Christopher S. Hlady, Gaurav Kanade, Sriram V. Pemmaraju, Philip M. Polgreen, Alberto M. Segre

**Affiliations:** 1 Department of Computer Science, The University of Iowa, Iowa City, Iowa, United States of America; 2 Department of Internal Medicine, The University of Iowa, Iowa City, Iowa, United States of America; Inserm & Universite Pierre et Marie Curie, France

## Abstract

We present a comprehensive approach to using electronic medical records (EMR) for constructing contact networks of healthcare workers in a hospital. This approach is applied at the University of Iowa Hospitals and Clinics (UIHC) – a 3.2 million square foot facility with 700 beds and about 8,000 healthcare workers – by obtaining 19.8 million EMR data points, spread over more than 21 months. We use these data to construct 9,000 different healthcare worker contact networks, which serve as proxies for patterns of actual healthcare worker contacts. Unlike earlier approaches, our methods are based on large-scale data and do not make any *a priori* assumptions about edges (contacts) between healthcare workers, degree distributions of healthcare workers, their assignment to wards, etc. Preliminary validation using data gathered from a 10-day long deployment of a wireless sensor network in the Medical Intensive Care Unit suggests that EMR logins can serve as realistic proxies for hospital-wide healthcare worker movement and contact patterns. Despite spatial and job-related constraints on healthcare worker movement and interactions, analysis reveals a strong structural similarity between the healthcare worker contact networks we generate and social networks that arise in other (e.g., online) settings. Furthermore, our analysis shows that disease can spread much more rapidly within the constructed contact networks as compared to random networks of similar size and density. Using the generated contact networks, we evaluate several alternate vaccination policies and conclude that a simple policy that vaccinates the most mobile healthcare workers first, is robust and quite effective relative to a random vaccination policy.

## Introduction

Healthcare-associated (or *nosocomial*) infections are a major cause of morbidity and mortality world-wide. The Centers for Disease Control and Prevention (CDC) estimate that 1.7 million people are directly affected by these infections every year [1]. A significant proportion of these infections, perhaps up to a third, are preventable [2]. Effective measures to control healthcare-associated infections include vaccinating healthcare workers (HCWs) against vaccine-preventable diseases, effective hand hygiene, restricting ill HCWs from patient care, environmental cleaning, and isolating patients infected or colonized with certain organisms (e.g., *Clostridium difficile*, methicillin-resistant *Staphylococcus aureus*) [3]–[6]. The effectiveness of these measures critically depends upon their implementation. For example, hand hygiene is thought to be the most effective way to prevent nosocomial infections, but less than 50% of HCWs practice adequate hand hygiene [7]–[10].

Improving the effectiveness of any infection control policy requires a clear understanding of how diseases spread within a hospital-based population. Through most of the 20th century, compartmental disease-spread models such as SIR (Susceptibe-Infected-Recovered) and its extensions [11] have provided analytical and computational tools for understanding the dynamics of disease spread in a relatively homogeneous population. These models are all based on the *mass-action principle* which posits that the number of new cases of disease in a small time interval is proportional to the product of numbers of infected and susceptible hosts in the previous time interval [12]. As Meyers points out [13], using the outbreak of SARS in China as an example, the mass-action assumption, when applied to a heterogeneous population, can lead to predictions of disease spread that are quite incompatible with the observed outbreak. *Contact network epidemiology* [13], [14] aims to overcome these limitations by explicitly modeling interactions between pairs of individuals as a network (graph) and studying the spread of disease through the population based on intrinsic features of the pathogen *and* structural properties of the network.

The use of contact network epidemiology to understand the spread of healthcare-associated infections within a large hospital has been quite limited, due mainly to the absence of reliable fine-grained data from which to infer contact networks that make epidemiological sense. There is now considerable research on the structure of online social networks (see for example [15]–[17]), but such online social networks are not always epidemiologically relevant and may be structurally very different from networks of HCWs induced by spatial and temporal proximity. Earlier work on contact network epidemiology in the hospital setting [18]–[21] start with limited data and used a number of modeling assumptions to construct contact networks. As a result, these approaches result in contact networks that are either highly structured (e.g., consisting of a clique for each ward or unit) or drawn at random from simple probability distributions. These types of networks do not seem representative of the complexity of interactions that occur in real hospital settings. More recent work (e.g., [22]) has moved away from modeling assumptions and has instead relied on fine-grained data obtained from the deployment of wireless sensor networks. The main advantage of a sensor-network-based approach is the resolution of the data: in the work of Isella et al. [22] active Radio-Frequency Identification Devices (RFID) achieve a 1.5 meter spatial resolution and a 20 second temporal resolution. Thus this type of data can be viewed as representing “ground truth,” rather than merely being a sample. However, due to high costs of deployment, significant privacy concerns for HCWs as well as patients, and concerns that the technology might interfere with normal hospital operations, these efforts are all limited in scale. For example, the work of Isella et al. cited above uses a week-long deployment in a pediatric ward and involves 119 participants. Other related work [23]–[25] has a similar time scale (ranging from 1 day to about 27 days) and size scale.

Our work relies on already-available data at a much larger scale; EMR logins that span 21 months, involve about 8,000 HCWs, and are spread over a 3.2 million square foot hospital with thousands of rooms. Besides scale and the relatively low cost of acquisition, another advantage of the EMR data relative to sensor-network-based approaches is the robustness of data. Devices in a sensor network suffer from issues such as failures, battery drainage, lack of time-synchronization, etc., and this can lead to a variety of errors that are hard to detect and account for. What we give up by using EMR data is the confidence that our data is a measure of “ground truth.” This is because our data only provides an indirect measure of proximity –- our definition of a “contact” is the event of two HCWs logging in to the EMR system in close spatio-temporal proximity. Nevertheless, we validate the HCW networks constructed via the EMR data using a 10-day long wireless sensor network deployment in the Medical Intensive Care Unit (MICU) at the UIHC [26]. Within the context of contact network epidemiology, healthcare-associated infections are being studied at different scales. Sensor network based data provides a fine-grained view, but usually at the level of a hospital unit. Other research [27]–[29] has focused at the regional level by using data on patient transfers within a regional hospital network. Our research occupies an important intermediate space between these two scales.

## Materials and Methods

### Electronic medical records

Like most modern U.S. hospitals, the UIHC has an Electronic Medical Record (EMR) system that HCWs regularly use to view and update patient records. Information required to care for patients is stored on the EMR system. For example, to learn the results of laboratory tests, recent vital signs, past medical history, medication histories, and allergies for a particular patient, a HCW must frequently access each patient's EMR. In addition, HCWs involved in patient care must log in to the EMR system to both read and update progress notes. This is true for both physicians and nurses. Physical therapists, occupational therapists and other consultants involved in the care of patients read notes generated by other HCWs and generate their own notes. Thus, because information about each patient is continually updated, a typical HCW caring for a specific patient needs to log into the EMR system using a terminal in close proximity to the patient during or just before or just after visiting that patient. There are more than 17,000 terminals available for HCW use, distributed throughout the hospital. For our purposes, each login into the EMR system by a HCW generates a record that is stamped with a time (login and logout times), a spatial location (a room in the UIHC), an anonymized ID corresponding to that HCW and a job type and department corresponding to the anonymized HCW. The UIHC logs staff access to the EMR system resulting in about 10 million login events per year. Table 1 shows the first five out of about 19.8 million de-identified records we were given access to. Aggregate characteristics of the entire EMR dataset are given in Table 2 showing the large size and diversity of individuals captured by the login data – 14,595 HCWs with 404 different job types spread over 80 departments. On any given day there are roughly 5,000 HCWs that login to the EMR.

**Table 1:** EMR Login Records.

login date & time	logout date & time	device^1^	location^2^	user ID	job type & department
2006-09-01, 0:00:00.40	2006-09-01, 0:24:17.29			A00012	STAFF NURSE I, NURSING
2006-09-01, 0:00:00.43	2006-09-01, 0:00:21.76	M95089	JPP 6750	A00029	STAFF NURSE II, NURSING
2006-09-01, 0:00:01.23	2006-09-01, 0:03:55.21			J00023	STAFF NURSE II, NURSING
2006-09-01, 0:00:02.29	2006-09-01, 0:00:14.81	MA1458	RCP 1100	C00112	HOUSE STAFF III, NEUROLOGY
2006-09-01, 0:00:02.54	2006-09-01, 0:46:37.82	B71118	RCP 1047	M00018	HOUSE STAFF I, ETC

The first five of approximately 19.8 million EMR login records. The userIDs are all de-identified, though they each have an associated jobtype & department field. Of the 19.8 million records, about 40% of the records are missing fields needed for contact network construction, still leaving about 11.7 million usable records.

^1^ Computer IDs with associated location information.

^2^ Rooms in the UIHC (e.g., RCP 1100 is room number 1100 in the Roy Carver Pavilion of the hospital).

**Table 2:** Aggregate characteristics of the EMR login data.

records	days	users	job types	departments	devices	locations
19.8 million	660	14,595	404	80	17,522	4,379

### The hospital graph

**Figure 1:**
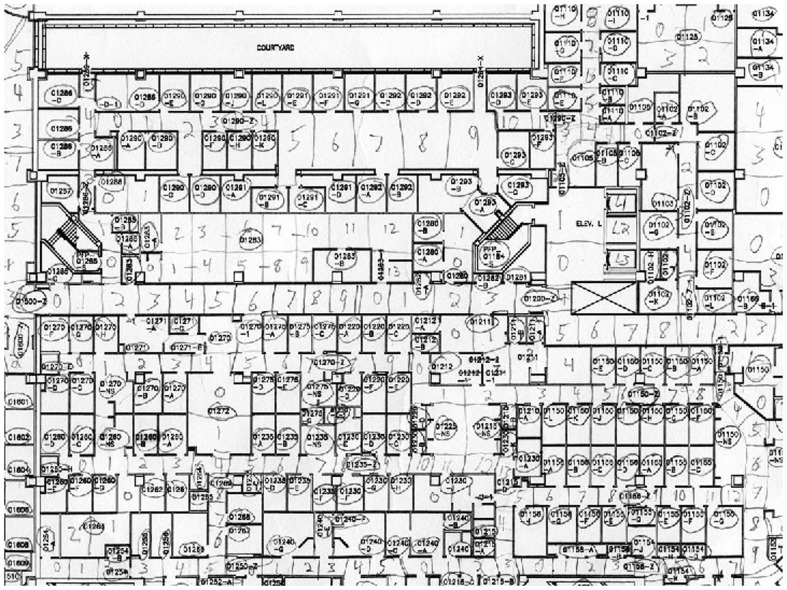
A marked up architectural CAD drawing fragment of the UIHC. This CAD drawing fragment corresponds to the basement (floor 0) of the hospital, showing how it was marked by hand in order to partition large open spaces and corridors into segments that were approximately room-sized.

From architectural blueprints of the UIHC facility we constructed, by hand, a *hospital graph* that provides a discrete model of the entire hospital space. Vertices in the hospital graph represent rooms (large open spaces and hallways are divided into “room-sized” chunks) and edges represent adjacencies (e.g., via doorways) between rooms (see Fig. 1). Aggregate statistics for the hospital graph are given in Table 3. The hospital graph essentially overlays a metric space (induced by pairwise hop-distances between hospital vertices) on the UIHC facility and allows us to precisely define the *mobility* of each HCW within a time window 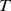 as the sum of the shortest path distances in the hospital graph between locations of consecutive logins that occur in 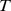. This provides us with a well-defined way of identifying “peripatetic” HCWs [30] and as we will show later, vaccinating these such HCWs is an effective strategy for reducing disease spread.

**Table 3:** Basic characteristics of the hospital graph.

vertices	edges	mean degree	diameter	mean path length
18,961	23,442	1.236	137	44.9

### Constructing HCW contact networks

Overlaying the EMR logins on top of the hospital graph provides fine-grained spatio-temporal coordinates for HCWs. We use these coordinates to infer HCW contact networks as follows. For non-negative integer parameters *d* and *t*, we say that a *contact* has occurred between two HCWs if they have logged in within *t* minutes of each other and within *d* hops of each other in the hospital graph. A more precise description of how the HCW contact networks are constructed follows.

Fix a time window 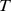 that corresponds to a contiguous sequence of days during the time period that we have EMR login data for. 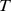 is 4 weeks long in all our analysis. Let 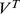 denote the set of users who have logged into the EMR system at least once during time window 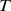. Fix integer parameters 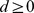 and 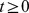. Each HCW 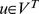 has a set 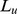 of login sessions that have occurred during time window 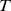, where each login session 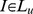 is defined by its *start time* 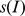, its *end time* 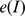, and its location or *placement* 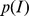. The placement 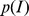 of a login event 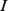 is a room (vertex) in the hospital graph. Two HCWs 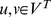 are connected by an edge if for some login sessions 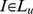 and 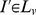, the distance in the hospital graph between 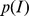 and 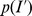 is at most 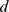 hops and the time interval 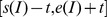 intersects the time interval 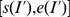. The edge 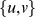 is assigned an edge-weight 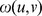 that is the number of login session pairs 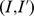 that satisfy the above conditions. Thus 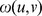 represents the number of distinct contacts between *u* and *v*, within the specified time window *T*, as indicated by their login records. Varying the values of *d* and *t* allows us to consider alternate notions of when a contact occurs. Specifically, as *d* and *t* increase, we essentially “loosen” the definition of a contact, thus producing denser contact networks. We also use an additional integer parameter 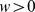 and drop from the edge set all edges with weight less than *w*. This allows us the flexibility of focusing on more consequential edges. Thus a HCW contact network is uniquely defined by the parameter 4-tuple 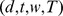. By varying *d* and *t* (thereby varying the notion of a contact) and by varying *T* and *w*, we construct 9,000 different HCW contact networks. Possible values of the parameters *d*, *t*, *w*, and *T* are described in Table 4.

**Table 4:** Parameters and their possible values for generating HCW contact networks.

Contact Network Generation Parameters	Possible Values
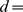 max. hop-distance between pairs of login locations	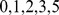
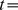 max. time (in minutes) between pairs of logins	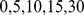
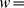 min. contacts between 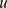 and 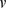 for 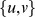 to exist	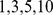
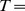 a 4-week time window	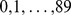

Time window 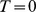 starts on 2006-09-03, 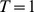 starts on 2006-09-10, etc. With 5 values for *d*, 5 for *t*, 90 for *T*, and 4 for *w*, all independently chosen, we obtain 9,000 different HCW contact networks.

**Note:** This research involves analysis of Electronic Medical Record accesses by healthcare workers at the University of Iowa Hospitals and Clinics. However, all of this data was anonymized before it reached us. In a memo dated 2/17/07, Dr. A. Bertolatus, M.D., chair of our IRB, ruled that the research described in our submission “did not meet the regulatory definition of human subjects research” and therefore “did not require review by the IRB” since we are “not collecting data on identifiable human subjects, nor collecting protected health care information.”

## Results

This section contains results from two types of analyses that we performed on HCW contact networks. First we evaluated structural characteristics of the HCW contact networks such as degree distribution, diameter, community structure, diversity by job type and diversity within job type, vulnerability to disease-spread, etc. Our analysis reveals that despite spatial and job-related constraints on HCW movement and interactions, there is a strong structural similarity between the HCW contact networks we generate and social networks that arise in other settings (e.g., movie or scientific collaborations, on-line friendships, etc. [15], [31]–[33]). Then we evaluate several alternate vaccination policies and conclude that a simple policy that vaccinates the most mobile HCWs first is robust and quite effective relative to a random vaccination policy. Our results provide a large-scale confirmation of the work of Temime et al. [30], who show the potential of highly mobile HCWs to cause “superspreading events.”

### Structural analysis of HCW contact networks

Table 5 shows statistics for the HCW contact networks we generate. As a convenient short hand, we use the names 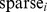, 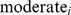, and 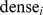 to denote the HCW contact networks with parameters 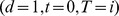, 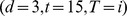, and 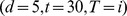 respectively. Where not explicitely noted we assume a threshold value of 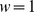. The resulting HCW contact networks exhibit many of the same structural properties that have been observed in social networks arising in other contexts such as the Karate club network [34], movie collaboration [33], scientific collaboration [31], e-mail network [32], and various online social networking services [15]. Specifically, all of the HCW contact networks have giant connected components that exhibit the *small-world property* [33] with all pairs of individuals having a “small degree of separation,” e.g., the average path length in the “giant component” of the 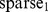 graph (with 5,838 vertices) is only 3.592. The graphs have a high *clustering coefficient* [33] with most pairs of neighboring individuals sharing a lot of contacts, e.g., the clustering coefficient of the 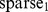 graph is about 1,000 times the clustering coefficient of the Erdös-Rényi random graph of same size and average degree. Tables S2 and S3 show that other HCW contact networks we construct also have very similar structural features.

**Table 5:** Basic structural features of HCW contact networks.

	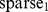	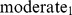	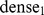
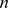 (num. vertices)	6,875	6,875	6,875
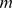 (num. edges)	82,199	174,739	332,766
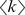 (mean degree)	23.91	50.83	96.8
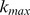 (max. degree)	321	635	1,115
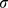 (std. dev. degree dist.)	32.84	62.86	113.877
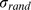 (std. dev. degree dist. 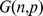 )	4.90	7.06	9.77
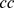 (clust. coeff.)	0.3109	0.3906	0.4379
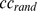 (clust. coeff. 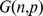 )	0.003516	0.007476	0.01414
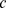 (num. components)	873	293	144
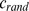 (num. components 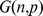 )	1	1	1
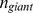 (num. vertices giant comp.)	5,838 (84.92%)	6,547 (95.23%)	6,702 (97.48%)
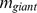 (num. edges giant comp.)	81,935 (99.68%)	174,687 (99.97%)	332,717 (99.98%)
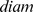 (diam. giant comp.)	11	13	12
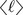 (ave. path len. giant comp.)	3.592	3.131	2.746

The 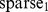, 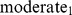, and 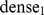 instances of the HCW contact network (4 weeks starting from Sept 10, 2006) are considered here. Note that the dense graph is only dense relative to the sparse graph; the average degree of even the dense graph is less than 1% of the complete graph size. For comparison, the corresponding statistics for Erdös-Rényi random graphs, 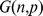, with same size (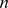) and same mean degree (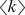) are also provided.

**Figure 2:**
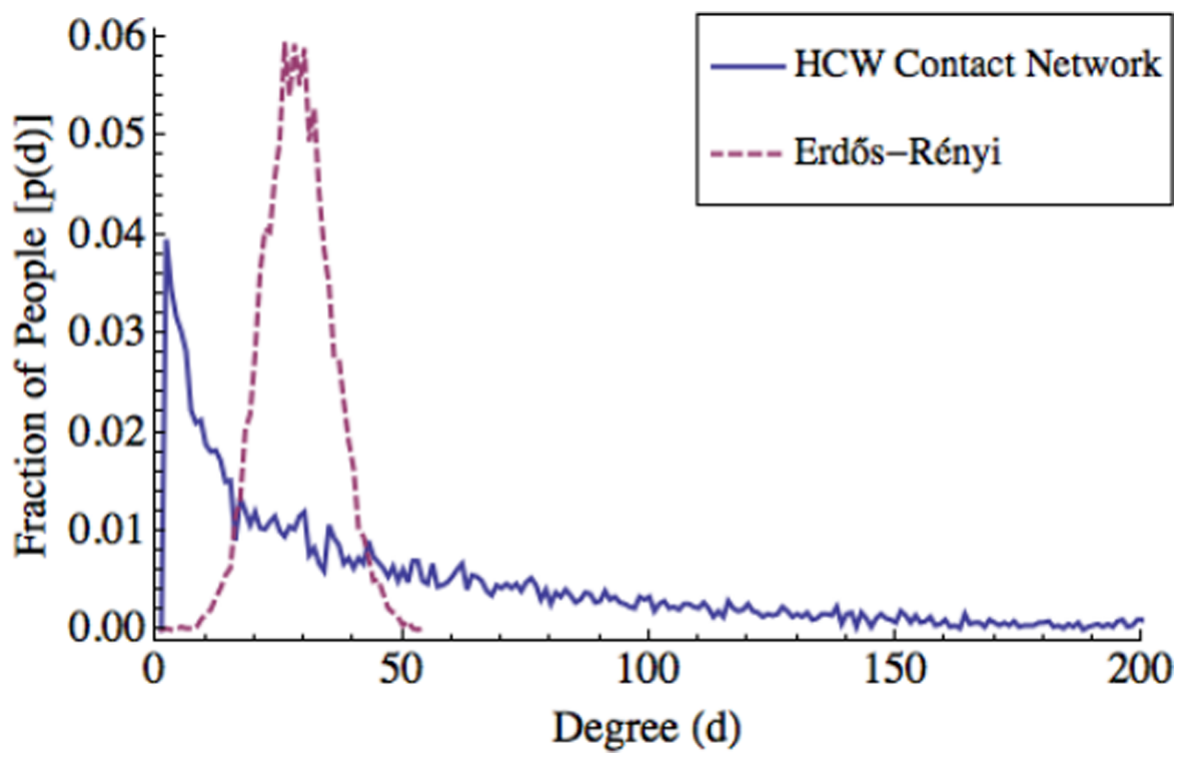
Degree distributions of the 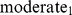 HCW contact network and corresponding Erdös-Rényi random graph. The Erdös-Rényi random graph has the same number of vertices and average degree as the 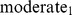 HCW contact network. The 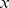-axis is truncated to 200, omitting 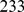 HCWs (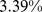) who make up the remainder of the heavy-tailed distribution. The maximum degree in the contact network is 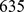.

HCW contact networks also exhibit a *heavy-tailed distribution* of contacts [35] with a few individuals having a large number of contacts and most individuals having very few. This differs significantly from the Poisson degree distribution of the Erdös-Rényi graphs (see Fig. 2) that is sharply concentrated about its mean. We present further analysis in the *Supporting Information*. Fig. S1(a) shows the log-log plot of the degree distribution of a moderate HCW contact network, indicating quite clearly that the distribution is heavy-tailed, covering close to three orders of magnitude and indicating a high level of heterogeneity among HCWs. Figs. S1(a) and (b) also show our attempts to fit the HCW contact network degree distribution to the popular heavy-tailed *power-law distribution* and *log-normal distribution* [36]. Visually, the log-normal seems to provide a reasonable fit, especially when viewing the cumulative density function plot (Fig. S1(b)), however, we also performed a Kolmogorov-Smirnov “goodness of fit” test (following the approach of Clauset et al. [37]) and obtained results that indicate that neither the power-law nor the log-normal are particularly good fits for the HCW contact network degree distributions. These results appear in Table S1. Even though specific well-known heavy tailed distributions do not explain the degree distribution of the HCW contact networks, the fact that the degree distribution is heavy tailed has important implications for infection control. If indeed a few people have lots of contacts, then it seems natural to try and target this group for vaccination.

Table 6 shows the categories of HCWs who contribute the most to the heavy tail (we use top 10%) of the degree distribution of the 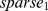 HCW contact network. The biggest contributers are Resident Physician (241) and Nurse (198) followed far behind by Physician (47), Therapist (24) and Nurse Assistant (20). It turns out that not only do lot of Resident Physicians have high degree in absolute numbers, but a large percentage of Resident Physicians (just over 40%) belong to the heavy tail. A much smaller percentage of Nurses (just over 10%) belong to the heavy tail.

**Table 6:** Who are the high degree nodes?

Job Category	Size of Top 10%	Size of Category	Percent in Top 10%
Resident Physician	241	595	40.50
Physician Assistant	14	42	33.33
Inpatient Unit Clerk	35	125	28
Nurse	198	1966	10.07
Physician	47	592	7.93
Therapist	24	328	7.31
Nurse Assistant	20	464	4.31
Misc. Patient Care Clerk	13	347	3.75
Misc. Patient Care	12	348	3.45

These are the categories of HCWs that contribute the most to the tail of the degree distribution of 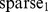 HCW contact network. We consider HCWs who fall in the top 10% by degree in 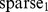 and identify job categories (shown in Column 1) that contribute at least 10 members to this group. Column 2 shows the contribution of each such job category. The job categories are sorted in decreasing order of the percentage of their members who belong to the high degree group.

Table 7 suggests that HCW contact networks have a strong *community structure*, i.e., a vertex-partition into densely connected groups, with few edges between groups. In particular, the table shows the *modularity* [38]–[40] for vertex-partitions obtained via four simple algorithms. Modularity values upwards of 0.3 suggest a strong community structure [41], [42]. The first two rows of the table correspond to vertex partitions of HCWs by “job type” and “department” respectively and these partitions have a low (i.e., relatively poor) modularity. This is to be expected because HCWs in the same job class (e.g., nurses) are widely dispersed across multiple departments, and departments are often composed of spatially dispersed units. The next two rows correspond to algorithms that yield vertex-partitions with strong community structure. The row labeled spatial partitions HCWs based on their “home location” (i.e., location of the machine into which a HCW logs in the most) in the hospital. The spatial algorithm for community partitioning is as follows. For each HCW 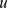, define a home location 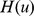 as the location of the computer in the hospital graph that 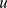 logs into most often. This maps each HCW onto a vertex in the hospital graph and moreover establishes a metric space on the set of HCWs with the distance between HCWs *u* and *v* being the hop distance in the hospital graph between 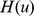 and 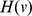, denoted 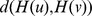. We then partition HCWs by making a graph where the nodes are all HCWs and an edge is placed between pairs of HCWs, *u* and *v*, if 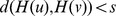 for some integer 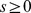. The connected components of this graph induce a partition of the healthcare workers. In our experiments we consider all possible values of 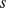 and find one (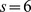) which maximizes the modularity of the community structure. The modularity values in the last row are obtained by using a “greedy” clustering algorithm, which we call maxQ, due to Clauset et al. [42]. The success of maxQ suggests that the HCW contact networks may contain a “hidden” community structure that is independent of job type, department, or even spatial attributes. This has important implications for infection control within a hospital, since it makes sense to focus resources on breaking links between communities, rather than on breaking up densely connected communities.

**Table 7:** Community structure of HCW contact networks.

	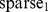	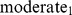	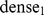
job type	.08	.05	.03
department	.21	.15	.12
spatial	.366	.312	.272
maxQ	.50	.38	.33

Modularity values for partitions of 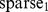, 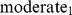, and 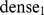 graphs obtained via different methods. The first row corresponds to partitioning HCWs by job type, the second corresponds to partitioning by department, the third row corresponds to an algorithm called spatial that clusters HCWs by their “home location” in the hospital, and the last row is obtained by using the maxQ algorithm (due to Clauset et al. [42]). Modularity values upwards of 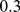 suggest strong community structure [41], [42].

**Figure 3:**
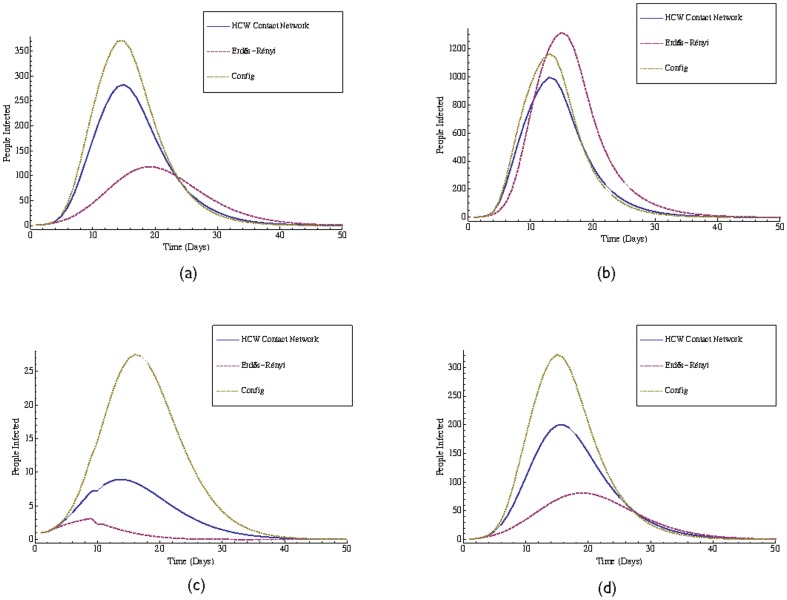
Disease-spread on HCW contact networks and on corresponding Erdös-Rényi and Config random graphs. (a) Plot for the 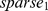 HCW contact network with threshold 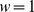 and the corresponding Erdös-Rényi and Config random graphs. (b) Plot for the 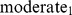 HCW contact network with threshold 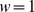 and the corresponding Erdös-Rényi random graph and Config random graphs. (c) Plot for the 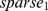 HCW contact networks with threshold 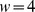 and the corresponding Erdös-Rényi random graph and Config random graphs. (d) Plot for the 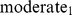 HCW contact networks with threshold 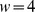 and the corresponding Erdös-Rényi random graph and Config random graphs. All plots show the number of infected individuals on each day over the lifetime of an infection that is initiated by a single randomly chosen individual. The solid (blue) curve is for HCW contact networks, the dashed (purple) curve is for Erdös-Rényi random graphs with size and average degree identical to the corresponding HCW contact network, and the dotted (tan) curve is for Config random graphs. Since the random graph models does not include a mechanism for modeling edge weights we give all edges uniform weight. The plots are obtained by using a disease-spread model that approximates the spread of influenza (see the *Materials and Methods* section for details of the SIR simulation that was used).

It is well known (see for e.g., [13], [14], [33]) that structural properties of contact networks such as those described above can have a significant effect on how disease spreads in a population. Fig. 3 compares the spread of disease on HCW contact networks with the spread of disease on “corresponding” Erdös-Rényi random graphs and the *Configuration* model random graphs (Config) [43]. For comparison, given an HCW contact network *G* with *n* vertices and average degree *d*, we generated an Erdös-Rényi random graph with *n* vertices and expected average degree equal to *d*. Similarly, we generated a Config graph that has the same degree distribution as *G*. Our simulations show that indeed, under conditions that approximate an influenza outbreak, disease-spread dynamics can be quite different on HCW contact networks relative to corresponding Erdös-Rényi and even Config networks. The Erdös-Rényi graphs display a “threshold” behavior in that as they become more dense, the number of infected people explodes. On the other hand the Config model consistently overestimates the number of infected people relative to the HCW network. This points to second-order effects (e.g., assortativity) that affect disease-spread, but are not modeled by Config [44]. The SIR simulation used to obtain these results is described next.

For our disease spread simulation we run a SIR-based model on our HCW contact networks. We assume that vaccination is 100% effective and thus individuals receiving vaccination are considered to be in the *Recovered* state. In our model, each individual is assumed to have the same susceptibility to disease, have the same infectiousness, remain sick the same amount of time, and stay active in the contact network for the entirety of the simulation. Infectivity is assumed to last for exactly *m* days. On the *i*th day of being infected, 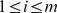, individual *j* spreads the disease to neighbor *k* with probability 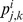. We chose to model influenza and use disease-spread parameters based on viral shedding levels provided by Carrat et al. [45]. We set 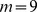 and set 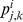 values according to the normalized vector of shedding levels 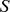 = (0.016645, 0.05, 0.035235, 0.02137, 0.011155, 0.007115, 0.005015, 0.003195, 0.00336) derived from plots in Carrat et al. Specifically, the *i*th entry in this vector, 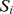 denotes the shedding level on day *i*. Then, we compute the 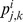 values using the formula 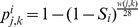. Recall that 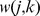 is the weight of edge 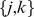, corresponding to the total number of contacts between *j* and *k* during time period *T* and therefore 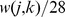 represents the average number of daily contacts between HCW *j* and *k* during a 4-week (28 day) period.

### Design of effective vaccination policies

**Figure 4:**
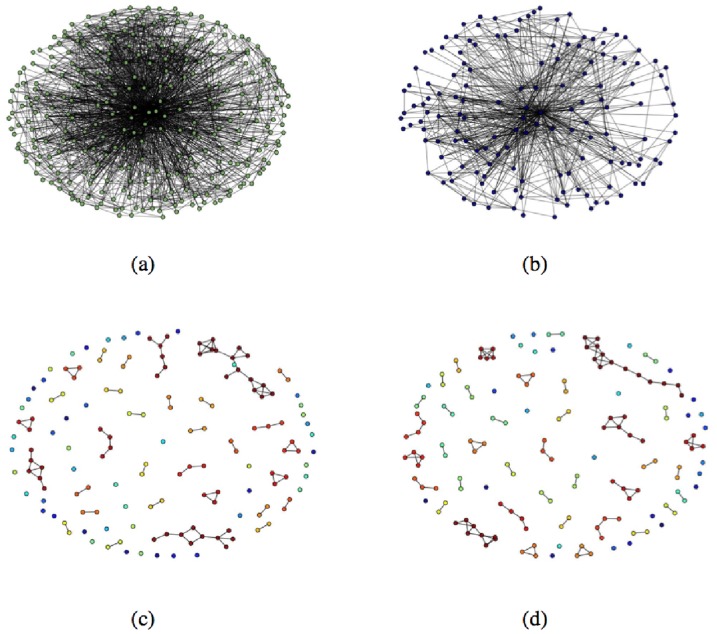
The effect of different vaccination policies on a HCW contact network. (a) Small portion of the 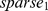 HCW contact network. The result of vaccinating 50% of the population using (b) the random policy, (c) the degree-based policy, and (d) the distance-based policy. The unvaccinated network in (b) consists of a single connected component, but in both (c) and (d) the HCW contact network is “shattered” into many tiny components.

**Figure 5:**
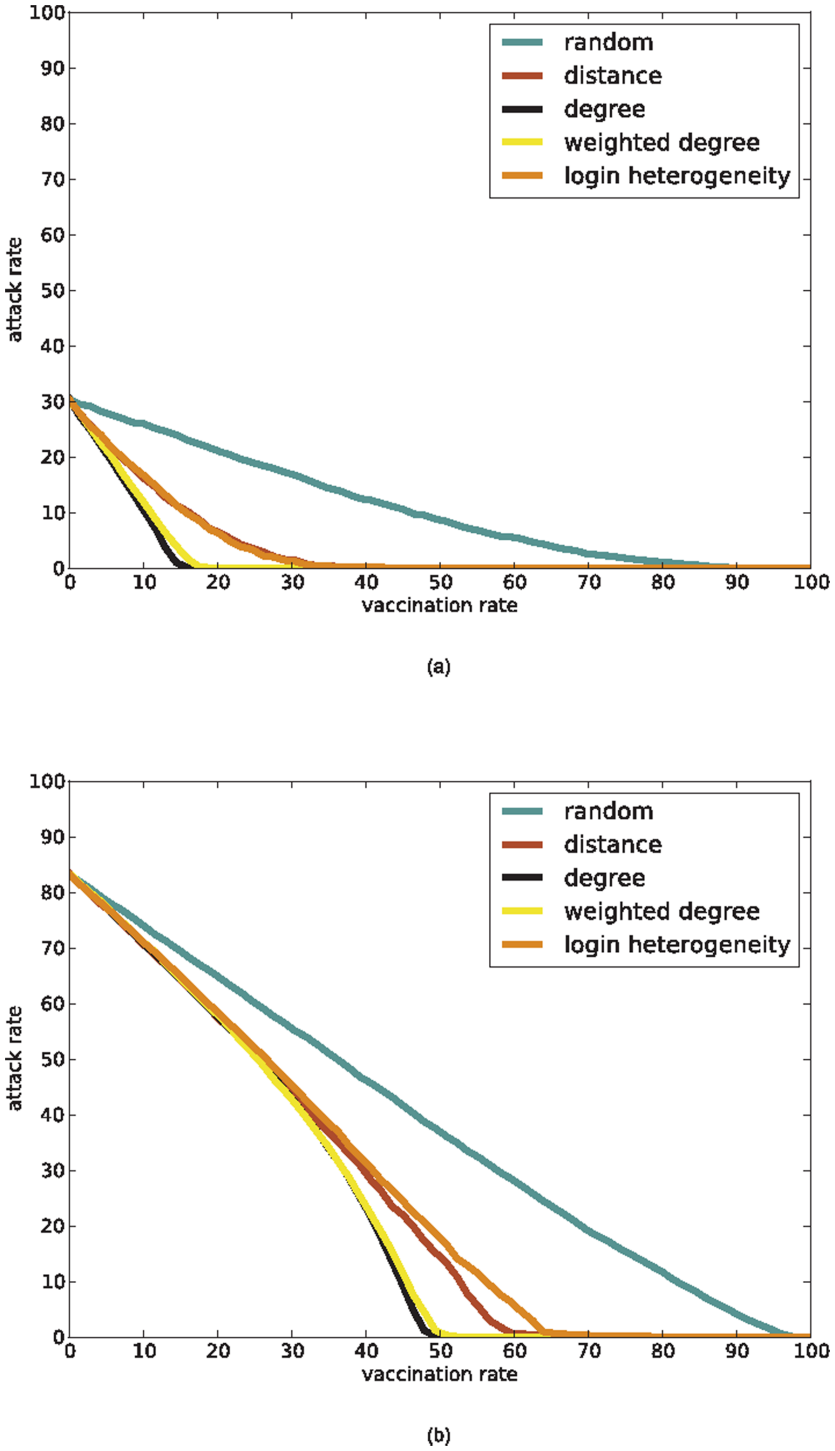
Effectiveness of vaccination policies on HCW contact networks. This effectiveness is measured by the expected number of people infected starting from a single infected individual chosen uniformly at random. We simulate an SIR-model that approximates the spread of influenza to produce these plots (see the *Materials and Methods* section for details). (a) All policies evaluated on the 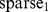 HCW contact network. The degree-based and weighted-degree-based policies are generated from this network; the distance-based and login-heterogeneity-based policies are generated from the EMR login data for 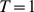. (b) All policies evaluated on the 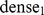 HCW contact network. The degree-based and weighted-degree-based policies are generated from this network; the distance-based and login-heterogeneity-based policies were generated from the EMR login data for 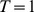.

Using the generated HCW contact networks we compare five different vaccination policies: (i) *random*, which vaccinates individuals picked uniformly at random; (ii) *degree-based*, which first vaccinates individuals with highest degree; (iii) *weighted-degree-based*, which first vaccinates individuals with highest *weighted degree*, defined as the sum of the weights of the edges incident on the individual; (iv) *distance-based*, which first vaccinates individuals with highest mobility; (v) *login-heterogeneity-based*, which first vaccinates individuals whose EMR logins have occurred at the most number of distinct computers. So two of our policies (degree-based and weighted-degree-based) depend on the “connectivity profile” of HCWs and two (i.e., distance-based and login-heterogeneity-based) depend on the “mobility profile” of HCWs. Since we assume that any vaccination that is administered is 100% effective and effective immediately, we model the action of vaccinating a person *v* as the deletion of the vertex *v* from the HCW contact network. Fig. 4 shows the effects of different policies on a HCW contact network. We evaluate the vaccination policies by computing the expected number of infected HCWs starting from a single infected individual chosen uniformly at random (Fig. 5). The plots suggest that the two connectivity-based policies are the best, followed by the two mobility-based policies. The findings on connectivity-based policies confirm results obtained by Christley et al. [46] on “small world” and “randomly mixing” graph models and by Bell et al. [47] on the spread of HIV on a network of cocaine injectors. Both connectivity-based and mobility-based policies are substantially better than the random policy. Further, the mobility-based policies approach the connectivity-based policies in effectiveness as the underlying HCW contact network becomes denser. The results for the mobility-based policies are in keeping with our expectation that highly mobile individuals are more likely to provide the “long distance” contacts that are critical for rapid disease spread [30], [33]. Fig. S2 shows that these plots remain essentially the same for different time windows *T*.

### Validation using sensor network data

In a recent paper [26], we showed that wireless sensor network data gathered from the Medical Intensive Care Unit (MICU) at the UIHC provides preliminary support for our assumption that HCW contact networks constructed from EMR login records are a reasonable proxy for actual physical proximity networks for HCWs. The reader is encouraged to see this paper for details of our sensor network deployment, characterestics of the gathered data, and results obtained from analyzing this data. Here we provide a brief sketch of the results from this paper.

We deployed a wireless sensor network in the MICU at the UIHC for a period of 10 days (June 1 to June 10, 2011). The deployed network consisted of fixed sensors (*beacons*) and wearable sensors (*badges*). On average, 20.1 badges were handed out to HCWs during day shifts and 13.2 during night shifts. All sensors emitted signals periodically, every 6 to 10 seconds. Proximity between pairs of HCWs and HCWs and fixed beacons was estimated using received signal strength. Beacons were distributed in hallways to help in triangulating HCW locations and they were also placed in every patient room (in the MICU) so that HCW visits to patient rooms could be detected.

Our overall approach to validating HCW contact networks is as follows. We take the sensor network data to be “ground truth” and construct *true* proximity networks in a standard manner (as in, for example [22]). We then construct a dataset of *synthetic logins* from the sensor network data using simple heuristic rules such as “a HCW who stays in a room for 4 minutes or longer must have accessed the patient's EMR data from the room's computer terminal.” From the synthetic logins dataset, we construct “proxy” HCW contact networks using the same method that we used to construct HCW contact networks from the EMR data. The main result of our analysis [26] is that the “proxy” HCW contact networks obtained from the synthetic login dataset are good predictors for the true proximity networks. In fact, in some cases the prediction obtained from the “proxy” HCW contact networks is better than predictions obtained by using standard link prediction algorithms.

## Discussion

### Diversity within subgroups

A closer analysis of the EMR login data and the HCW contact networks shows that there is great deal of diversity even within groups of HCWs belonging to the same department and having the same job type. For example, the degree and mobility distributions of the ten largest HCW groups (see Table S4) by department and job type all exhibit a heavy-tailed distribution (see Fig. S3). This observation highlights the importance of large-scale data for constructing HCW networks and the fact that approaches that take subgroups of HCWs within the same job type or department to be homogeneous may yield contact networks that are not representative.

### Limitations of our approach

We are aware of several problems with using EMR login data to constructing HCW contact networks. First of all, EMR login events are simply a sample of spatio-temporal locations of HCWs and the fundamental question one might ask about our approach is whether this sample is good enough. A preliminary, positive answer to this question is provided by our use of data from the wireless sensor network deployment in the MICU to validate HCW contact networks. However, our validation itself suffers from a few limitations. For one, HCW movement and login patterns at the MICU might be quite different from those in other UIHC units and as a result, our preliminary positive results at the MICU may not carry over to other units. A second limitation arises from our approach of generating synthetic logins. While we have used simple, intuitive rules to generate synthetic logins, these may themselves differ in structure from actual logins. We plan on addressing these problems in the future by (i) doing wireless sensor network deployments in other units and (ii) seeking EMR login data that overlaps in time with our deployment.

It is also worth pointing out that even though our sensor network deployment provides preliminary validation of our use of EMR logins to generate HCW contact networks, it does not provide conclusive guidance with regards to which combination of 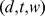 parameters are most appropriate from an epidemiology point of view. In fact, it is quite possible that different parameter settings are appropriate in different hospital units, due to differences in login patterns and placement of terminals in different units. We plan to address this issue also with further sensor network deployments.

Other problems with our EMR login-based approach include the absence of patients and certain categories of HCWs who don't regularly access the EMR system (e.g., janitors, transporters, etc). Visitors are also not present in our data set. We have started to analyze data acquired recently from the UIHC on patient admissions and discharge and out-patient load and this analysis will lead to models that will help populate our contact networks with patients.

### Modeling vaccination policies and their effectiveness

**Figure 6:**
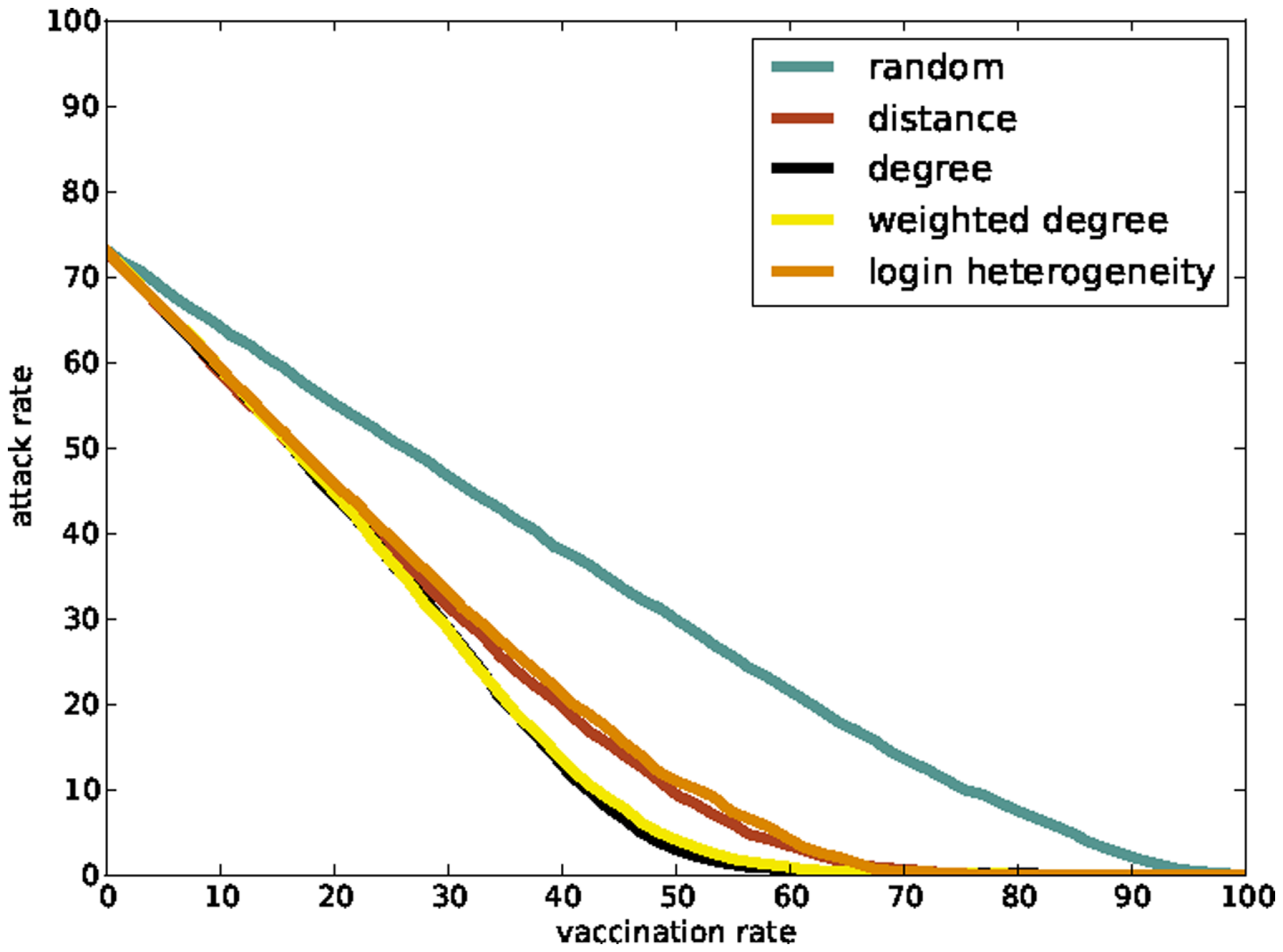
Effectiveness of vaccination policies on a “time-shifted” HCW contact network. The connectivity-based policies are generated from the 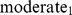 HCW contact network. The mobility-based vaccination policies are also generated using the EMR login data for 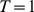. The plot shows the effectiveness of these policies on the “time-shifted” HCW contact network, 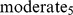, measured by the expected number of people infected starting from a single infected individual chosen uniformly at random. The 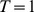-network and the 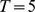-network not only differ in edges, but also in the HCWs they contain as vertices.

Fig. 5 seems to imply that the connectivity-based policies (i.e., degree-based and weighted-degree-based) are more effective than mobility-based policies (i.e., distance-based and login-heterogeneity-based). However, the experiments in these figures give the connectivity-based policies an unfair advantage by evaluating them on the very same networks that they were generated from. A more realistic evaluation would generate connectivity-based policies on a particular HCW contact network, but evaluate these on a different, but structurally similar network. Since we have many HCW contact networks at our disposal, such an evaluation is easy and is shown in Fig. 6. This plot suggests that mobility-based policies are as effective as connectivity-based policies when the HCW contact networks used to generate the policies only approximately represent actual contact patterns. Furthermore, individual mobility is easier and cheaper to track than even simple HCW contact network characterestics of an individual such as degree.

## Conclusions

We present a comprehensive approach to constructing and using HCW contact networks in hospitals. This can be applied at any hospital that records access to EMR logins. The utility of constructing HCW contact networks goes well beyond modeling disease spread and designing control policies. HCW contact networks can be used to solve problems in health-care optimization [48] including the placement of resources critical for health-care delivery and the architectural design (or redesign) of hospital units. HCW contact networks can also be used to model and study “peer effects” within HCWs that seem to influence the adoption of effective medical practices (e.g., regular hand hygiene, timely completion of medical records, vaccine uptake) within a hospital setting [49], [50].

## Supporting Information

Figure S1 **Fitting power-law and log-normal distributions to the HCW contact network degree distribution.**
(PDF)

Figure S2 **Vaccination policies have the same effect on HCW contact networks from different time periods.**
(PDF)

Figure S3 **HCWs within the same job category are quite diverse.**
(PDF)

Table S1 **Results from a Kolmogorov-Smirnov “goodness of fit” test.**
(PDF)

Table S2 **Structural features of moderate HCW contact networks for different time windows.**
(PDF)

Table S3 **Structural features of the moderate_1_ HCW contact networks with low weight edges dropped.**
(PDF)

Table S4 **Sizes of different categories of HCWs.**
(PDF)

## References

[1] Klevens R, Edwards J, Richards C, Horan T (2007) Estimating health care-associated infections and deaths in US hospitals, 2002. Public Health 122: 160–166. 17357358 10.1177/003335490712200205 PMC1820440

[2] Haley R, Culver D, White J, Morgan W, Emori T, et al (1985) The efficacy of infection surveillance and control programs in preventing nosocomial infections in US hospitals. Am J Epidemiol 121: 182–205. 4014115 10.1093/oxfordjournals.aje.a113990

[3] Edmond M, Wenzel R (2005) Organization for Infection Control. In: Mandell G, Bennett J, Dolin R, editors, Principles and Practice of Infectious Diseases, 6th ed, Philadelphia, PA: Churchill Livingstone. p. 3323.

[4] Garner J, Simmons B (1983) Guideline for isolation precautions in hospitals. Infect Control 4: 245–325. 6309693

[5] Garner J (1996) Guideline for isolation precautions in hospitals. Part I. Evolution of isolation practices. Am J Infect Control 24: 24–31. 8651517 10.1016/s0196-6553(96)90050-4

[6] Garner J (1996) Guideline for isolation precautions in hospitals. Infect Control Hosp Epidemiol 17: 53–80. 8789689 10.1086/647190

[7] Boyce J, Pittet D (2002) Guidelines for Hand Hygiene in Health-Care Settings: Recommendations of the Healthcare Infection Control Practices Advisory Committee and the HICPAC/SHEA/APIC/IDSA Hand Hygiene Task Force Guidelines for Hand Hygiene in Health-Care Settings: Recommendation. Infection Control and Hospital Epidemiology 23: S3–S41. 12515399 10.1086/503164

[8] Haas J, Larson E (2007) Measurement of Compliance with Hand Hygiene. J Hospital Infec 66: 6–14. 10.1016/j.jhin.2006.11.013 17276546

[9] Kampf G, Löffler H, Gastmeier P (2009) Hand hygiene for the prevention of nosocomial infections. Deutsches Ärzteblatt international 106: 649–655. 19890431 10.3238/arztebl.2009.0649 PMC2770229

[10] Pittet D, Hugonnet S, Harbarth S, Mourouga P, Sauvan V, et al (2000) Effectiveness of a hospitalwide programme to improve compliance with hand hygiene. Infection Control Programme. Lancet 356: 1307–1312. 11073019 10.1016/s0140-6736(00)02814-2

[11] Hethcote HW (2000) The mathematics of infectious diseases. SIAM Review 42: 599–653.

[12] Kermack WO, McKendrick AG (1927) A contribution to the mathematical theory of epidemics. Proc Roy Soc Lond A 115: 700–721.

[13] Meyers L (2007) Contact network epidemiology: Bond percolation applied to infectious disease prediction and control. Bulletin: American Mathematical Society 44: 63–86.

[14] Newman MEJ (2002) The spread of epidemic disease on networks. Physical Review E 66: 016128. 10.1103/PhysRevE.66.016128 12241447

[15] Ahn YY, Han S, Kwak H, Moon S, Jeong H (2007) Analysis of topological characteristics of huge online social networking services. In: Proceedings of the 16th international conference on World Wide Web (WWW). pp. 835–844.

[16] Mislove A, Marcon M, Gummadi KP, Druschel P, Bhattacharjee B (2007) Measurement and analysis of online social networks. In: Proceedings of the 7th ACM SIGCOMM conference on Internet measurement (IMC). pp. 29–42.

[17] Kleinberg J (2008) The convergence of social and technological networks. Communications of the ACM 51: 66–72.

[18] Bernard H, Fischer R, Mikolajczyk R, Kretzschmar M, Wildner M (2009) Nurses' contacts and potential for infectious disease transmission. Emerg Infect Dis 15: 1438–1444. 19788812 10.3201/eid1509.081475 PMC2819878

[19] Meyers L, Newman M, Martin M, Schrag S (2003) Applying network theory to epidemics: control measures for mycoplasma pneumoniae outbreaks. Emerging Infectious Diseases 9: 204–210. 12603991 10.3201/eid0902.020188 PMC3369603

[20] Polgreen P, Tassier T, Pemmaraju S, Segre A (2010) Prioritizing healthcare worker vaccinations on the basis of social network analysis. Infection Control and Hospital Epidemiology 31: 893–900. 20649412 10.1086/655466 PMC3024853

[21] Ueno T, Masuda N (2008) Controlling nosocomial infection based on structure of hospital social networks. Journal of Theoretical Biology 254: 655–666. 18647609 10.1016/j.jtbi.2008.07.001 PMC7094152

[22] Isella L, Romano M, Barrat A, Cattuto C, Colizza V, et al (2011) Close encounters in a pediatric ward: Measuring face-to-face proximity and mixing patterns with wearable sensors. PLOS One 6. 10.1371/journal.pone.0017144 PMC3046133 21386902

[23] Stehlé J, Voirin N, Barrat A, Cattuto C, Colizza V, et al (2011) Simulation of an SEIR infectious disease model on the dynamic contact network of conference attendees. BMC Medicine 9. 10.1186/1741-7015-9-87 PMC3162551 21771290

[24] Olguín DO, Gloor PA, Pentland AS (2009)Wearable sensors for pervasive healthcare management. In: 3d International Conference on Pervasive Computing Technologies for Healthcare. pp. 1–4.

[25] Kazandjieva MA, Lee JW, Salathé M, Feldman MW, Jones JH, et al. (2010) Experiences in measuring a human contact network for epidemiology research. In: ACM Workshop on Hot Topics in Embedded Networked Sensors (HotEmNets).

[26] Herman T, Monsalve M, Pemmaraju S, Polgreen P, Segre AM, et al. (2012) Inferring realistic intra-hospital contact networks using link prediction and computer logins. In: 2012 ASE/IEEE International Conference on Social Computing and 2012 ASE/IEEE International Conference on Privacy, Security, Risk and Trust. pp. 572–578.

[27] Donker T, Wallinga J, Grundmann H (2010) Patient referral patterns and the spread of hospitalacquired infections through national health care networks. PLoS Comput Biol 6: e1000715. 20333236 10.1371/journal.pcbi.1000715 PMC2841613

[28] Lesosky M, McGreer A, Simor A, Green K, Low D, et al (2011) Effect of patterns of transferring patients among healthcare institutions on rates of nosocomial methicillin-resistant staphylococcus aureus transmission: a monte carlo simulation. Infect Control Hosp Epidemiol 32: 136–147. 21460468 10.1086/657945

[29] Liljeros F, Giesecke J, Holme P (2007) The contact network of inpatients in a regional healthcare system. a longitudinal case study. Mathematical Population Studies 14: 269–284.

[30] Temime L, Opatowski L, Pannet Y, Brun-Buisson C, Bolle PY, et al (2009) Peripatetic healthcare workers as potential superspreaders. Proceedings of the National Academy of Sciences 106: 18420–18425. 10.1073/pnas.0900974106 PMC2775324 19841263

[31] Barabasi A, Jeong H, Neda Z, Ravasz E, Schubert A, et al (2002) Evolution of the social network of scientific collaborations. Physica A 311: 590–614.

[32] Kossinets G, Watts DJ (2006) Empirical Analysis of an Evolving Social Network. Science 311: 88–90. 16400149 10.1126/science.1116869

[33] Watts D, Strogatz S (1998) Collective dynamics of ‘small-world’ networks. Nature 393: 440–442. 9623998 10.1038/30918

[34] Zachary WW (1977) An information flow model for conflict and fission in small groups. Journal of Anthropological Research 33: 452–473.

[35] Barabasi AL (2003) Linked: How Everything Is Connected to Everything Else and What It Means for Business, Science, and Everyday Life. Plume Books

[36] Mitzenmacher M (2003) A brief history of generative models for power law and lognormal distributions. Internet Mathematics 1.

[37] Clauset A, Shalizi CR, Newman MEJ (2009) Power-law distributions in empirical data. SIAM Review 51: 661–703.

[38] Girvan M, Newman ME (2002) Community structure in social and biological networks. Proc Natl Acad Sci U S A 99: 7821–7826. 12060727 10.1073/pnas.122653799 PMC122977

[39] Newman M, Girvan M (2004) Finding and evaluating community structure in networks. Phys Rev E 69: 026113. 10.1103/PhysRevE.69.026113 14995526

[40] Newman M (2004) Detecting community structure in networks. Europen Physical Journal B 38: 321–330. 10.1103/PhysRevE.69.066133 15244693

[41] Newman MEJ (2004) Fast algorithm for detecting community structure in networks. Physical Review E 69: 066133. 10.1103/PhysRevE.69.066133 15244693

[42] Clauset A, Newman MEJ, Moore C (2004) Finding community structure in very large networks. Phys Rev E 70: 066111. 10.1103/PhysRevE.70.066111 15697438

[43] Molloy M, Reed B (1998) The size of the giant component of a random graph with a given degree sequence. Combinatorics, Probability, and Computing 7: 295–306.

[44] Newman M (2002) Assortative mixing in networks. Physical Review Letters 89: 208701. 12443515 10.1103/PhysRevLett.89.208701

[45] Carrat F, Vergu E, Ferguson NM, Lemaitre M, Cauchemez S, et al (2008) Time lines of infection and disease in human influenza: A review of volunteer challenge studies. American Journal of Epidemiology 167: 775–785. 18230677 10.1093/aje/kwm375

[46] Christley R, Pinchbeck G, Bowers R, Clancy D, French N, et al (2005) Infection in social networks: Using network analysis to identify high-rist individuals. American Journal of Epidemiology 162: 1024–1031. 16177140 10.1093/aje/kwi308

[47] Bell D, Atkinson J, Carlson J (1999) Centrality measures for disease transmission networks. Social Networks 21: 1–21.

[48] Curtis D, Hlady C, Pemmaraju S, Polgreen P, Segre A (2010) Modeling and estimating the spatial distribution of healthcare workers. In: IHI 2010: 1st ACM International Health Informatics Symposium. In press.

[49] Curtis D, Hlady C, Pemmaraju S, Segre A, Polgreen P (2010) Social network influence on vaccination uptake among healthcare workers. In: 5th Decennial International Conference on Healthcare-Associated Infections.

[50] Curtis D, Pemmaraju S, Polgreen L, Polgreen P, Segre A (2010) Peer effects and influenza vaccination among healthcare workers. In: 3rd Biennial Conference of the American Society of Health Economists.

