# Supplementary material for: Healthcare Worker Contact Networks and the Prevention of Hospital-Acquired Infections
Source: PLoS One. 2013 Dec 30;8(12):e79906. doi: 10.1371/journal.pone.0079906 (PMC3875421; doi:10.1371/journal.pone.0079906)
Supplement: Figure S1 — Fitting power-law and log-normal distributions to the HCW contact network degree distribution. (PDF) [file pone.0079906.s001.pdf]

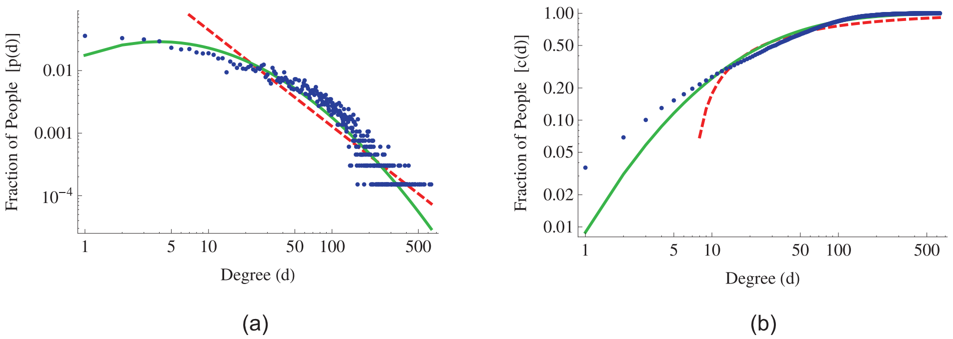

Figure 1: **Fitting power-law and log-normal distributions to the HCW contact network degree distribution.** (a) The log-log plot of the degree distribution of the $\texttt{moderate}_1$ HCW contact network. Each point $(d, p(d))$ represents the fraction $p(d)$ of HCWs with degree d in the $\texttt{moderate}_1$ HCW contact network. The plot also shows the maximum likelihood power-law (red, dashed line) and log-normal (green, solid curve) fits to the degree distribution. (b) The same degree distribution, but in its cumulative form (each point $(d, c(d))$ represents the fraction $c(d)$ of HCWs with degree at most d) is shown here. Corresponding maximum likelihood power-law (red, dashed curve) and log-normal fits (green, solid curve) are also shown.
