# Supplementary material for: Healthcare Worker Contact Networks and the Prevention of Hospital-Acquired Infections
Source: PLoS One. 2013 Dec 30;8(12):e79906. doi: 10.1371/journal.pone.0079906 (PMC3875421; doi:10.1371/journal.pone.0079906)
Supplement: Figure S2 — Vaccination policies have the same effect on HCW contact networks from different time periods. (PDF) [file pone.0079906.s002.pdf]

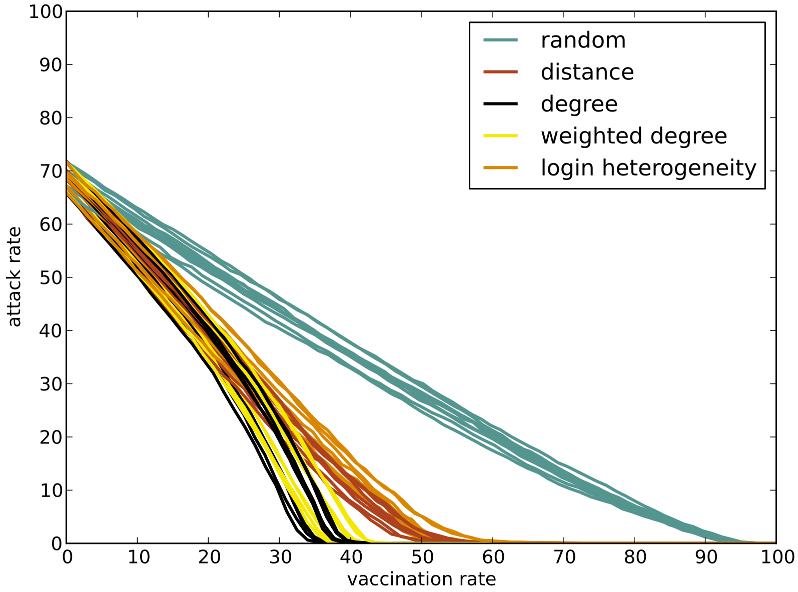

Figure 2: **Vaccination policies have the same effect on HCW contact networks from different time periods.** Effectiveness of different vaccination policies on different $\texttt{moderate}_i$ HCW contact networks as measured by the size of the largest connected component in the unvaccinated network. Plots for HCW contact networks from 9 different four-week time windows are shown. Specifically, we use $T = 1, 2, 8, 9, 50, 51, 52, 85, 86$.
