# Supplementary material for: Healthcare Worker Contact Networks and the Prevention of Hospital-Acquired Infections
Source: PLoS One. 2013 Dec 30;8(12):e79906. doi: 10.1371/journal.pone.0079906 (PMC3875421; doi:10.1371/journal.pone.0079906)
Supplement: Figure S3 — HCWs within the same job category are quite diverse. (PDF) [file pone.0079906.s003.pdf]

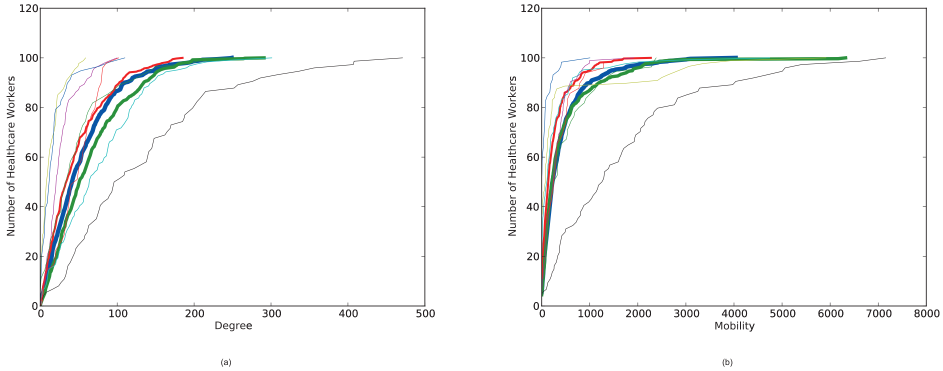

Figure 3: **HCWs within the same job category are quite diverse.** Cumulative density plots for the (a) degree distribution and the (b) mobility distribution for each of the largest 10 groups of HCWs partitioned by (department, job title) pairs. Both plots are based on EMR login data for time window $T = 1$ and the degree distribution is calculated using on the The plots show, for a particular degree (respectively, mobility) d, the fraction of HCWs within a group whose degree (respectively, mobility) is at most d.
