# Supplementary material for: Healthcare Worker Contact Networks and the Prevention of Hospital-Acquired Infections
Source: PLoS One. 2013 Dec 30;8(12):e79906. doi: 10.1371/journal.pone.0079906 (PMC3875421; doi:10.1371/journal.pone.0079906)
Supplement: Table S1 — Results from a Kolmogorov-Smirnov “goodness of fit” test. (PDF) [file pone.0079906.s004.pdf]

Table 1: **Results from a Kolmogorov-Smirnov "goodness of fit" test.**

power-law fit					
Graph type	x_{min}	α	% disc	p-value	p-value
$\texttt{sparse}_1$	4	1.56	24.49	0	0
$\texttt{moderate}_1$	7	1.54	19.69	0	0
$\texttt{dense}_1$	12	1.53	16.59	0	0

log-normal fit				
Graph Type	μ	σ	p-value	p-value
$\texttt{sparse}_1$	2.54	1.36	0	0.14
$\texttt{moderate}_1$	3.25	1.37	0	0.03
$\texttt{dense}_1$	3.90	1.37	0	0

Columns 2-3 of the two tables respectively show the parameters of the power-law distribution and the log-normal distribution obtained using the Maximum Likelihood Estimator method. The proportion of discarded samples (i.e., those that are less than x_{min}) is indicated in Column 4 of the power-law table. After selecting the optimal values of the parameters for both distributions, we test whether the provided model of the data can be accepted or not. The p-values of the Kolmogorov-Smirnov test are shown in the last 2 columns of the two tables. Following Clauset et al. [xx] we use the p-value as a measure of the hypothesis we are trying to verify (rather than the null hypothesis), and hence high values, not low, are "good." The second last column in each table shows p-values obtained via comparing against a large number of synthetically generated data sets (a method described by Clauset et al. [xx]). The last column in each table was obtained by looking up Table 9.30 on page 570 of "Probability and Statistics" by DeGroot and Schervish (Addison Wesley, Boston, 2002). The values are based on a result due to Kolmogorov and Smirnov.
