# Supplementary material for: Healthcare Worker Contact Networks and the Prevention of Hospital-Acquired Infections
Source: PLoS One. 2013 Dec 30;8(12):e79906. doi: 10.1371/journal.pone.0079906 (PMC3875421; doi:10.1371/journal.pone.0079906)
Supplement: Table S2 — Structural features of moderate HCW contact networks for different time windows. (PDF) [file pone.0079906.s005.pdf]

Table 2: **Structural features of moderate HCW contact networks for different time windows.**

	$T = 20$	$T = 50$	$T = 80$
n (num. vertices)	6,960	7,144	7,330
m (num. edges)	179,525	191,270	220,662
$\langle k \rangle$ (mean degree)	51.59	53.55	60.22
k_{max} (max. degree)	601	660	724
σ (std. dev. degree dist.)	63.736	64.704	72.314
σ_{rand} (std. dev. degree dist. $G(n,p)$)	7.24	7.34	7.87
cc (clust. coeff.)	0.3521	0.4131	0.3979
cc_{rand} (clust. coeff. $G(n,p)$)	0.007437	0.007425	0.008170
c (num. components)	345	323	295
c_{rand} (num. components $G(n,p)$)	1	1	1
n_{giant} (num. vertices giant comp.)	6,565 (94.32%)	6,770 (94.76%)	6,954 (94.87%)
m_{giant} (num. edges giant comp.)	179,450 (99.96%)	191,196 (99.96%)	220,468 (99.91%)
$diam$ (diam. giant comp.)	11	11	13
$\langle \ell \rangle$ (ave. path len. giant comp.)	3.030	3.134	3.030

Basic structural features of moderate HCW contact networks, $\texttt{moderate}_{20}$, $\texttt{moderate}_{50}$, and $\texttt{moderate}_{80}$. The time windows $T = 20, 50, 80$ correspond to 4-week period starting on January 21st, 2007, August 19th, 2007, and March 16th, 2008, respectively. Statistics for all of these different time windows are very similar to those shown for the $\texttt{moderate}_{1}$ HCW contact network in Table 4. For comparison, the corresponding statistics for Erdös-Rényi random graphs, $G(n,p)$, with same size (n) and same mean degree ($\langle k \rangle$) are also provided.
