# Supplementary material for: Healthcare Worker Contact Networks and the Prevention of Hospital-Acquired Infections
Source: PLoS One. 2013 Dec 30;8(12):e79906. doi: 10.1371/journal.pone.0079906 (PMC3875421; doi:10.1371/journal.pone.0079906)
Supplement: Table S3 — Structural features of the moderate1 HCW contact networks with low weight edges dropped. (PDF) [file pone.0079906.s006.pdf]

Table 3: **Structural features of the $\texttt{moderate}_1$ HCW contact networks with low weight edges dropped.**

	$w = 1$	$w = 4$	$w = 9$
n (num. vertices)	6,875	6,875	6,875
m (num. edges)	174,739	61,393	28,709
$\langle k \rangle$ (mean degree)	50.83	17.86	8.35
k_{max} (max. degree)	635	226	122
σ (std. dev. degree dist.)	62.86	24.36	13.714
σ_{rand} (std. dev. degree dist. $G(n,p)$)	7.06	4.22	2.88
cc (clust. coeff.)	0.3906	0.3445	0.2919
cc_{rand} (clust. coeff. $G(n,p)$)	0.007476	0.002596	0.001123
c (num. components)	293	1224	2506
c_{rand} (num. components $G(n,p)$)	1	1	1
n_{giant} (num. vertices giant comp.)	6,547 (95.23%)	5,570 (81.01%)	4,251 (61.83%)
m_{giant} (num. edges giant comp.)	174,687 (99.97%)	61,288 (99.83%)	28,547 (99.44%)
$diam$ (diam. giant comp.)	13	11	15
$\langle \ell \rangle$ (ave. path len. giant comp.)	3.131	3.911	4.781

Basic structural features of the $\texttt{moderate}_1$ HCW contact networks for threshold values of $w = 1, 4, 9$. Even though the contact networks become more sparse as the threshold w increases, the networks retain their properties. For example, even for $w = 9$ the network has high clustering coefficient (0.2919 versus 0.001123 for corresponding Erdös-Rényi graphs). Similarly, the average path length is only 4.781 even when $w = 9$.
