# Supplementary material for: Healthcare Worker Contact Networks and the Prevention of Hospital-Acquired Infections
Source: PLoS One. 2013 Dec 30;8(12):e79906. doi: 10.1371/journal.pone.0079906 (PMC3875421; doi:10.1371/journal.pone.0079906)
Supplement: Table S4 — Sizes of different categories of HCWs. (PDF) [file pone.0079906.s007.pdf]

Table 4: **Sizes of different categories of HCWs.**

**department**	**job title**	**group size**
NURSING	STAFF NURSE II	795
NURSING	STAFF NURSE I	617
NURSING	NRS ASST	378
NURSING	NRS UNIT CLK	124
RESPIRATORY CARE	RESP THERAPIST	94
PATHOLOGY	CL LAB SCI II	88
INTERNAL MEDICINE	HSE STAFF FELL	74
NURSING	PSY NUR ASST I	58
INTERNAL MEDICINE	PROFESSOR	55
RADIOLOGY	IMAGING TECHN	49

The largest ten groups of HCWs that logged in during time window $T = 1$ partitioned by distinct (department, job title) pairs. 664 out of 6875 HCWs (9.65%) had no assigned department or job title in the data we obtained.
